# Clinical factors associated with quality of life among women with endometriosis: a cross-sectional study

**DOI:** 10.1186/s12905-023-02694-5

**Published:** 2023-10-24

**Authors:** Karin Pontoppidan, Matts Olovsson, Hanna Grundström

**Affiliations:** 1 Department of Women’s and Children’s Health, Uppsala University, Uppsala, Sweden; 2 Department of Obstetrics and Gynaecology in Norrköping, Department of Biomedical and Clinical Sciences, Linköping University, Linköping, Sweden; 3 Department of Health, Medicine and Caring Sciences, Linköping University, Linköping, SE - 581 83 Sweden

**Keywords:** Endometriosis, EHP-30, Endometriosis Health Profile-30, Quality of life, Patient-centeredness

## Abstract

**Background:**

Endometriosis often leads to a decrease in Quality of Life (QoL), due to its impact on various aspects of women’s lives, such as social life, mental health, sex life, and working capacity. Although previous studies have assessed QoL in women with endometriosis, few studies have explored the impact of different clinical variables on QoL. The aim of this study was to investigate how women with endometriosis perceive their QoL, and to analyze which clinical factors are associated with QoL.

**Methods:**

The Endometriosis Health Profile-30 and the ENDOCARE Questionnaire were distributed to 1000 women diagnosed with endometriosis from 10 different clinics across Sweden. The responses from 476 women were included in univariate and multivariable regression analyses, where the clinical factors were correlated with overall QoL and QoL dimensions.

**Results:**

The women participating in this study reported a low QoL. The clinical factors that showed a significant correlation with overall QoL were age at first onset of endometriosis symptoms (β= -0.64, p < 0.001), having more than 10 visits to general practitioners before referral to a gynecologist (β = 5.58, p = 0.036), current or previous mental health issues (β = 7.98, p < 0.001) patient-centeredness (β= -2.59, p < 0.001) and use of opioids (β = 7.14, p = 0.002).

**Conclusions:**

This study shows that opioid use and mental health issues were associated with a worse QoL, whereas a higher degree of patient-centeredness was associated with a better QoL. The association between opioid use and a worse QoL might not entirely be caused by the opioid use itself but also by symptom severity and mental health issues. An improved patient-centeredness and more focus on taking care of mental health issues would reasonably result in a better QoL for women with endometriosis.

## Background

Endometriosis is a chronic disease that affects approximately 10% of women during their reproductive years [1, 2]. The condition is characterized by the presence of endometrium-like tissue outside the uterine cavity, such as in the ovaries, pelvic peritoneum, or rectum [3]. Common symptoms of endometriosis include dysmenorrhea, chronic pelvic pain, dyspareunia, infertility, dysuria, and dyschezia, although 20–25% of women with endometriosis are asymptomatic [4, 5]. It often takes several years to be correctly diagnosed, with reported diagnostic delays ranging from 5 to 10 years [6–8].

Endometriosis often has a negative impact on Quality of Life (QoL) as it affects several aspects of women’s lives, including social life, mental health, sex life, and working capacity [9–11]. Previous studies have mainly focused on symptom severity and how factors such as dysmenorrhea, infertility and pelvic pain affect QoL. Pessoa et al. found that the symptoms with the most negative impact on QoL are heavy periods, pelvic pain, dysmenorrhea and dyspareunia. Patients reporting more symptoms, or higher experienced severity of their disease, had lower QoL in all measured aspects [12]. Having endometriosis and pelvic pain, particularly non-menstrual pelvic pain, is associated with a lower QoL and poorer mental health, with more symptoms of depression and anxiety compared to asymptomatic endometriosis patients, as well as healthy controls [13]. Furthermore, younger women experience more symptoms of endometriosis than older women and have a worse perceived QoL [10].

Whether clinical factors such as having a responsible gynecologist to care for endometriosis, a plan for regular follow-ups, diagnostic delay, and experienced level of patient-centeredness, may affect QoL thus remains unknown. It is crucial to investigate the potential impact of different clinical variables on QoL in women with endometriosis since a better understanding can form a basis for targeted improvement of endometriosis care. Therefore, the aim of this study was to investigate how women with endometriosis perceive their QoL, and to analyze which clinical factors are associated with QoL.

## Methods

### Study design

This is a cross-sectional study conducted on a national sample of women with diagnosed endometriosis. Participants were recruited from three university hospitals, five county hospitals, and two district hospitals in Sweden.

### Data collection

A contact person from each of the participating clinics obtained a list of social security numbers for 150 women aged 18 and older who were diagnosed with endometriosis (ICD-10 codes N80.1-N80.9) and had visited the clinic during the last five years due to endometriosis-related symptoms. From each list, 100 women were randomly selected and invited to participate in the study via an invitation letter including patient information, a link, and a QR code to access the digital survey. The survey included clinical and sociodemographic questions as well as validated instruments to measure QoL and patient-centeredness. Women who chose to participate provided informed consent by completing the survey, and a reminder was sent to those who did not respond within three weeks. Data was collected in September 2021.

### Endometriosis health profile-30 (EHP-30)

The EHP-30 is an instrument used to measure QoL, consisting of 30 items divided into 5 different dimensions: Pain (11 items), Control and Powerlessness (6 items), Emotional Well-being (6 items), Social Support (4 items) and Self-Image (3 items). The items are answered on a 5-point Likert scale. The scores in each dimension generate a sum score ranging from 0 to 100, where a higher score indicates a worse QoL. Each sum score is calculated by using the following formula: Sum of scores for each item in the dimension / (4 (maximum score per item) x no. of items in the dimension). Besides the sum scores for each dimension, an overall score for the five dimensions combined is calculated using the same formula.

The EHP-30 is considered a valid, reliable, and disease-specific instrument [14, 15] that has been translated, cross-culturally adapted [16], and psychometrically evaluated in a Swedish context with good results and a Cronbach’s α 0.83–0.96 [17].

### Endocare questionnaire (ECQ)

The ECQ is an instrument that allows women to rate their experience with different organizational aspects of healthcare and grade the importance of these aspects. It can be used to compare patient-centeredness between different clinics and to identify targets for improvement [18]. ECQ is a valid, reliable, and disease-specific instrument that has been translated, cross-culturally adapted, and psychometrically evaluated in a Swedish context with good results [19].

The instrument consists of two parts. The first part includes 21 questions about background data, such as age, education, employment status, and endometriosis-related symptoms. The second part consists of 38 statements about different situations and aspects of the care the woman receives. She grades her agreement with the statements and the importance of the aspect on two 4-point Likert scales [20]. The ECQ generates various outcome measures, which have been described in previous studies [20]. This study will focus on the patient-centeredness score, which takes into account the performance/experience and its importance and is graded from 0 to 10, where a higher score indicates more patient-centeredness.

### Data analysis

From EHP-30, a total QoL sum score was calculated, and sum scores for each of the 5 different dimensions. The total patient-centeredness score was calculated from the 10 dimensions of the ECQ. Missing answers were omitted from the calculations. Demographic data and clinical variables were presented as frequency and percentage for nominal data, and mean and standard deviation for interval data.

To identify clinical risk factors for low QoL, both univariate linear regression and multiple regression analyses were conducted. In the univariate regressions, clinical factors were assessed for their association with total QoL scores. Clinical factors with a p-value less than 0.2 [21] in the univariate regression were selected for further analysis in the multiple regression analysis, using the enter model building method, to evaluate their independent effect on QoL. Nominal variables with more than two categories were dichotomized. To assess the degree of multicollinearity between any of the factors in the multiple regression, variance inflation factor (VIF) was evaluated for each factor. A VIF of more than 5 indicates that there is a considerable multilinearity between any of the factors [22].

The following clinical factors were included in the regression analysis: ‘age at first symptoms of endometriosis’, ‘diagnostic delay’ (time from symptom onset to diagnosis), ‘endometriosis severity’, ‘>10 visits to general practitioners before referral to a gynecologist’, ‘having a responsible gynecologist to care for endometriosis’, ‘ever tried to conceive for > 12 months’, ‘previous or current mental health issues’, ‘usage of hormonal treatment’, ‘usage of opioids’, and ‘patient-centeredness’. The variable regarding number of visits to general practitioners before referral to a gynecologist was dichotomized using the cutoff value of > 10 as the answers ranged between 0 and 1000. The variable regarding severity was dichotomized as minimal/mild and moderate/severe.

The clinical factor ‘usage of opioids’ was created by reviewing all free-text answers to determine which pain medications were used. ‘Patient-centeredness’ was determined by calculating the total patient-centeredness score from the ECQ. The other clinical factors included in the analysis were derived from answers in the first part of the ECQ.

The level of significance was set to p < 0.05. Regression coefficients (β) represent the mean change in the outcome variable (total EHP-30 score) for every 1-unit change in the independent variable (the clinical factor). The explained variance of the multiple regression model was presented with adjusted R^2^. The data analysis was performed in IBM SPSS Statistics version 28.

## Results

A total of 476 women completed the digital survey, resulting in a response rate of 48%. Background information and clinical factors are presented in Table 1. The mean age was 36.5 years, and the mean diagnostic delay, defined as the time from symptom onset to diagnosis, was 9.4 years. 24% of the women reported having visited general practitioners more than 10 times before referral to a gynecologist. Around two-thirds of the women had a treatment plan and a responsible gynecologist to care for their endometriosis. Hormonal treatment was used by 73% of the women, 74% received pain medication of any type and 26% were currently using opioids to some extent, regularly or on demand. The total patient-centeredness score was 3.7 on average.

**Table 1:** Demographic data and clinical factors

Parameters		
Age, years, mean ± SD	36.5 ± 9.0	
Swedish as Native Language, *n* (% of valid answers)		
Yes		441 (92.8)
No		34 (7.2)
Higher Education, *n* (% of valid answers)		
Yes		262 (55.2)
No		213 (44.8)
Working full time, *n* (% of valid answers)		
Yes		250 (52.5)
No		226 (47.5)
Age at First Symptoms, years, mean ± SD		19.4 ± 7.7
Delay, years, mean ± SD		
Patient’s Delay		3.3 ± 4.7
Doctor’s Delay		6.5 ± 6.9
Diagnostic Delay		9.4 ± 7.6
No. of Visits to General Practitioners before referral, median, (IQR) >10 Visits to General Practitioners before referral, *n* (% of valid answers)		5, 9 103 (24)
Treatment Plan, *n* (% of valid answers)		
Yes		301 (63.2)
No		175 (36.8)
Responsible Gynecologist for Endometriosis, *n* (% of valid answers)		
Yes		315 (66.2)
No		161 (33.8)
Ever Tried to Conceive > 12 months, *n* (% of valid answers)		
Yes		163 (34.3)
No		213 (65.7)
Has Children, *n* (% of valid answers)		
Yes		240 (50.5)
No		235 (49.5)
Previous or Current Mental Health Issues, *n* (% of valid answers)		
Yes		262 (55.6)
No		209 (44.4)
Hormonal Treatment, *n* (% of valid answers)		
Yes		346 (72.7)
No		130 (27.3)
Pain Medication, *n* (% of valid answers)		
Yes		352 (74.4)
No		121 (25.6)
Usage of Opioids		
Yes		125 (35.6)
No		226 (64.4)
Disease Severity, *n* (% of valid answers)		
Minimal/Mild		73 (26.4)
Moderate/Severe		204 (73.6)
Total Patient-Centeredness Score, mean ± SD		3.7 ± 1.9

The results from the EHP-30 are presented in Table 2. The average total score for all dimensions was 45.9. The best QoL was found in the ‘pain’ dimension, with a mean score of 36.7 ± 26.4, where lower scores indicate a better QoL. The worst QoL was found in the ‘control and powerlessness’ dimension, with a mean score of 51.2 ± 31.0.

**Table 2:** Sum scores from the QoL-instrument Endometriosis Health Profile 30 (EHP-30), for each dimension and in total

EHP-30 Dimension	Sum Score, Mean ± SD
Pain	36.7 ± 26.4
Control and Powerlessness	51.2 ± 31.0
Emotional Wellbeing	42.4 ± 23.0
Social Support	48.9 ± 28.8
Self-Image	48.1 ± 30.9
Total	45.9 ± 24.5

Table 3 presents the results from the univariate linear regression analyses, where each clinical factor was correlated with overall QoL and QoL dimensions. The results showed that ‘age at first symptoms of endometriosis’, ‘diagnostic delay’, ‘>10 visits to general practitioners before referral to a gynecologist, ‘previous or current mental health issues’, ‘usage of opioids’ and ‘patient-centeredness’ were significantly associated with overall QoL. However, ‘endometriosis severity’, ‘having a responsible gynecologist to care for endometriosis’, ‘ever tried to conceive for > 12 months’, and ‘usage of hormonal treatment’ were found to be non-significant, and were therefore excluded from the subsequent multivariate linear regression analysis.

**Table 3:** Regression Coefficients (β) and *p-*values for the univariate regression analysis between each determinant and quality of life dimensions, and overall quality of life

	Overall		Pain		Control and powerlessness		Emotional well-being		Social Support		Self-Image	
Age at first symptoms of endometriosis	-1.05;	< 0.001*	-0.88	< 0.001*	-1.22;	-1.01	-1.01	< 0.001*	-1.29	< 0.001*	-1.01	< 0.001*
Diagnostic delay	0.44	0.004*	0.35	0.028*	0.57	0.40	0.40	0.133*	0.62	< 0.001*	0.40	0.039*
Endometriosis severity (moderate/severe)	2.45	0.486	2.18	0.567	3.36	4.35	4.35	0.652	3.56	0.376	4.35	0.304
> 10 visits to GP before referral to a gynecologist	16.71	< 0.001*	17.45	< 0.001*	18.19	20.26	20.26	< 0.001*	17.51	< 0.001*	20.26	< 0.001*
Having a responsible gynecologist to care for endometriosis	2.75	0.256*	4.01	0.118	5.02	2.97	2.97	0.565	0.82	0.773	2.97	0.322
Ever tried to conceive for > 12 months	-0.77	0.750	-1.93	0.449	1.15	-1.63	-1.63	0.464	-0.94	0.740	-1.63	0.586
Previous or current mental health issues	13.21	< 0.001*	8.99	< 0.001*	12.98	16.12	16.12	< 0.001*	15.06	< 0.001*	16.12	< 0.001*
Usage of hormonal treatment	-1.06	0.679*	-3.87	0.156	0.161	-2.04	-2.04	0.616	-0.40	0.893	-2.04	0.522
Usage of opioids	12.14	< 0.001*	17.22	< 0.001*	15.90	10.30	10.30	0.003*	11.09	< 0.001*	10.30	0.001*
Patient-centeredness	-4.39	< 0.001*	-4.30	< 0.001*	-5.03	-3.96	-3.96	< 0.001*	-5.10	< 0.001*	-3.96	< 0.001*

*= *p* < 0.2 GP = general practitioner

The results for the QoL dimensions were similar to the associations with overall QoL, with some exceptions. ‘Having a responsible gynecologist to care for endometriosis’ was significantly associated with the ‘control and powerlessness’ and ‘pain’ dimensions, and ‘usage of hormonal treatment’ was significantly associated with the ‘pain’ dimension.

The multivariate linear regression results are presented in Table 4, which shows that several factors were independently associated with overall QoL. The adjusted R^2^ for the model was 0.34, meaning that 34% of the variance in overall QoL scores was explained by the clinical factors included in the model. The VIF was less than 5 for all variables, suggesting that there was no considerable multicollinearity between any of the factors [22].

**Table 4:** Regression Coefficients (β) and significant *p-*values (< 0.05) for the multivariable regression analysis between determinants and quality of life dimensions, and overall quality of life

	Overall		Pain		Control and powerlessness		Emotional well-being		Social Support		Self-Image	
Age at first symptoms of endometriosis	-0.64;	< 0.001	-0.56;	0.006	-0.76	< 0.001	-0.46	0.013	-0.75	< 0.001	-0.58	0.026
Diagnostic delay	NS		-0.42	0.033	NS		-0.35	0.048	NS		NS	
> 10 visits to GP before referral to a gynecologist	5.58	0.036	NS		NS		NS		NS		9.17	0.015
Having a responsible gynecologist to care for endometriosis			7.73	0.007	7.80	0.015						
Previous or current mental health issues	7.98	< 0.001	NS		NS		9.21	< 0.001	9.53	< 0.001	12.63	< 0.001
Usage of hormonal treatment			-6.70	0.021								
Usage of opioids	7.14	0.002	10.87	< 0.001	10.05	0.001	NS		5.74	0.045	NS	
Patient-centeredness	-2.59	< 0.001	-3.20	< 0.001	-3.40	< 0.001	-1.81	0.002	-3.60	< 0.001	-1.81	0.030

GP = general practitioner, NS = not significant

Previous or current mental health issues was associated with worser overall QoL (β = 7.98, p < 0.001), and this was the factor with the strongest association with QoL. Further, usage of opioids was associated with significantly worse overall QoL (β = 7.14, p = 0.002), as was lower patient-centeredness (β= -2.59, p < 0.001). Having >10 visits to general practitioners before referral was also associated with a worse overall QoL (β = 5.58, p = 0.036). Additionally, younger age at first symptoms of endometriosis was associated with worse overall QoL (β= -0.64, p < 0.001). The only non-significant clinical factor in the multiple regression against overall QoL was diagnostic delay.

Regarding the QoL dimensions, using opioids was associated with worse outcomes in the ‘pain’ (β = 10.87, p < 0.001), ‘control and powerlessness’ (β = 10.05, p < 0.001) and ‘social support’ (β = 5.74, p = 0.045) dimensions. Having previous or current mental health issues was associated with worse outcomes in the ‘emotional wellbeing’ (β = 9.21, p < 0.001), ‘social support’ (β = 9.53, p < 0.001) and ‘self-image’ (β = 12.63, p < 0.001) dimensions respectively. Further, having a responsible gynecologist to care for endometriosis was associated with worse outcomes in the ‘pain’ (β = 7.73, p = 0.007) and ‘control and powerlessness’ (β = 7.8, p = 0.015) dimensions, while diagnostic delay was associated with worse outcomes in the ‘pain’ (β=-0.42, p = 0.033) and ‘emotional wellbeing’ (β=-0.35, p = 0.048) dimensions. Having > 10 visits in primary care before referral was only associated with worse outcome in the ‘self-image’ (β = 9.17, p = 0.015) dimension, and usage of hormonal treatment was associated with better outcome in the ‘pain’ dimension (β =-6.70, p = 0.021). Finally, lower patient centeredness and younger age at first symptoms of endometriosis were associated with worse outcomes in all dimensions.

## Discussion

To the best of our knowledge, this is the first study to use an endometriosis-specific instrument to measure QoL and assess how clinical factors affect QoL in women with endometriosis. In comparison with previous studies [4, 23] the women in our study reported worse overall QoL. One possible explanation is that our study population was more burdened by their endometriosis and endometriosis related symptoms. Previous research has linked symptom severity to worse QoL [4], and in our study, 73.6% reported their endometriosis as severe. However, our study’s reported severity levels were not higher than those reported in other studies [7, 23, 24]. This suggests that symptom severity alone cannot fully account for the low QoL observed in our study population, and the reason behind this low QoL cannot be fully explained.

A prior study found that anxiety and depression were associated with worse QoL [21], which is consistent with our results. Mental health issues was also the factor that showed the strongest correlation with overall QoL in our sample. When analyzing each dimension separately, mental health issues affected the ‘emotional wellbeing’, ‘social support’ and ‘self-image’ dimensions significantly. This makes sense, as mental health issues are tightly connected to emotional wellbeing, and there are known associations between social support and protection from depression [25]. Overall, our results suggest that mental health issues affect various aspects of QoL among women with endometriosis, and further studies are needed to assess this relation in more detail.

Another clinical factor that showed a strong correlation to QoL was the usage of opioids. More specifically, there were significant associations between using opioids and scoring worse in the ‘pain’, ‘control and powerlessness’ and ‘social support’ dimensions. Scoring worse in the ‘pain’ dimension suits well since women who are prescribed opioids typically experience more pain, and pelvic pain has been shown to decrease QoL in other studies [4, 13]. More than a quarter of our study population reported using opioids, which could indicate that our population was heavily burdened by the disease, or that doctors in Sweden are generally liberal when it comes to prescribing opioids for treating pain related to endometriosis. Currently, the ESHRE guidelines as well as the national Swedish guidelines recommend restrictive use of opioids when treating pain related to endometriosis [26, 27]. Meanwhile, many patients with endometriosis experience severe pain that reduces their daily function, which is also an important issue to address. Additionally, it can be hypothesized that if opioid use reflects a heavier disease burden, it might consecutively result in a reduced sense of control. That could explain the association between having opioids prescribed and scoring worse in the ‘control and powerlessness’ dimension.

Having a responsible gynecologist to care for endometriosis was significantly correlated to the ‘pain’ and ‘control and powerlessness’ dimensions. It can be speculated that only the more severe cases of endometriosis have a responsible gynecologist, and that these women experience more pain than the average endometriosis population. Similar to the arguments about opioid use, having a responsible gynecologist might also reflect a higher disease burden, and thereby a diminished sense of control over your situation. Yet, a recent study identified having a responsible gynecologist to care for treatment and follow-up as the single most important factor for experiencing high patient-centeredness [28].

A lower age at first symptoms of endometriosis was associated with a worse QoL. Earlier research has shown that younger women with endometriosis tend to experience more symptoms [9] and report lower satisfaction with care [22], which may partially explain why younger age at endometriosis debut was associated with a lower QoL. However, disease debut may be less relevant to current perceived QoL than other factors.

A higher degree of patient-centeredness was associated with a better QoL, which is consistent with previous qualitative studies showing the importance of patient-centered factors for women’s health [9]. Apers et al. also investigated the correlation between patient-centered endometriosis care (PCEC) and QoL, using the ECQ and EHP-30 instruments. They found a correlation between the PCEC dimension continuity and overall QoL, as well as between overall PCEC and the EHP-30 dimension of social support [23]. These findings are in line with our results, which also demonstrated a correlation between patient-centeredness and QoL.

In our study population, 52.5% of the women had a higher education, which is similar to the Swedish population of the same gender and age [29]. Additionally, our study population was selected from 10 different clinics across Sweden, which suggests that our population is representative of the national population of women with endometriosis. This is a notable strength of our study.

The response rate of 47.6% in our study is considered acceptable. We were able to obtain a large sample size, allowing for more precise conclusions that can be applicable to the general endometriosis population. Additionally, a strength of our study is that only women with diagnosed endometriosis were included. In some other studies, self-reported endometriosis is used, making it more difficult to interpret the results for the endometriosis population.

Since the study was conducted in Sweden, the results may not be generalizable to populations in other countries, as healthcare systems and cultural factors may differ. Furthermore, our study design only allows for correlations to be drawn, and not causality. Therefore, it is not possible to determine whether the factors identified in our study directly influence QoL or are simply associated with it. As with all self-reported surveys, there may be a risk of response bias, as women may be hesitant to report sensitive information or may not fully understand the questions being asked. Finally, another limitation of this study is the possibility of recall bias, as some answers to questions may be more difficult to recall accurately than others, which could impact the validity of the results. For example, both diagnostic delay and the number of visits to general practitioners before referral to a gynecologist are factors that can be affected by recall bias, as it requires the woman to remember events that may have happened many years ago.

## Conclusion

Several clinical factors showed a significant correlation with QoL, especially mental health issues, opioid prescription, and patient-centeredness. Mental health issues and opioid prescription was associated with a worse QoL, whereas a higher degree of patient-centeredness was associated with a better QoL. The association between opioid use and a worse QoL might not entirely be caused by the opioid use itself but also by symptom severity and mental health issues. An improved patient-centeredness and more focus on taking care of mental health issues would reasonably result in a better QoL for women with endometriosis.

## Data Availability

The dataset analysed during the current study is not publicly available due to ethical reasons but are available from the corresponding author on reasonable request.

## Abbreviations

ECQ: EndoCare Questionnaire
EHP-30: Endometriosis Health Profile-30
ESHRE: European Society for Human Reproduction and Embryology
PCEC: Patient–Centered Endometriosis Care
QoL: Quality of Life
SF-36: Short Form Health Survey-36
VIF: Variance Inflation Factor
